# The X-Ray Crystal Structures of Primary Aryl Substituted Selenoamides

**DOI:** 10.3390/molecules14020884

**Published:** 2009-02-23

**Authors:** Yang Li, Guo-Xiong Hua, Alexandra M. Z. Slawin, J. Derek Woollins

**Affiliations:** School of Chemistry, University of St Andrews, St Andrews, Fife, Scotland, KY16 9ST, UK

**Keywords:** Woollins reagent, Primary selenoamides, X-ray structures

## Abstract

The X-ray structures of 12 primary selenoamides are reported. Metric parameters are provided, together with an illustration of the range of hydrogen bonding motifs.

## Introduction

**Figure 1:**
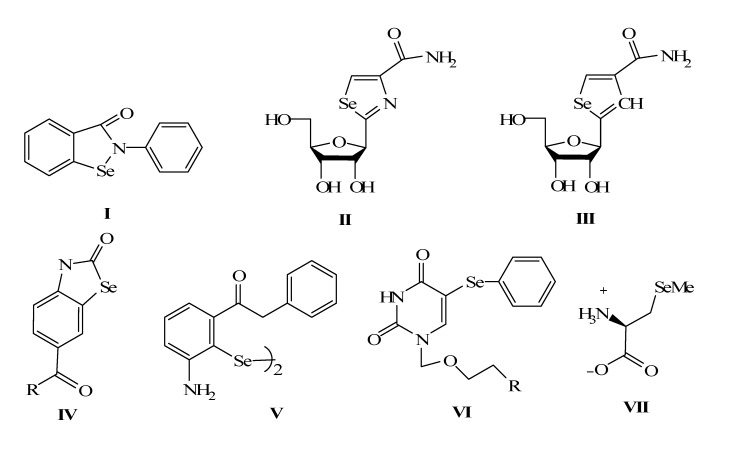
Some biologically active selenium compounds.

Selenium is an essential element for life; e.g, selenocysteine is recognised as the 21st amino acid and the importance of selenium containing enzymes in redox processes has is now recognized [1,2,3]. Many organoselenium compounds have been studied as biological models that simulate catalytic functions demonstrated by natural enzymes [4,5,6,7,8,9,10,11,12,13,14]. For example, ebselen (**I**, Figure 1) acts as a glutathione peroxidase (GP_x_) mimic and as a scavenger of peroxinitrite and the activity of the sulfur analogue of ebselen was 15-fold lower than that of ebselen [5,6]. Selenazofurin (**II**) has been reported to be a potent inhibitor of phlebovirus infections [10]. Selenophenfurin (**III**) exhibits antiproliferative and inosine 5^’^-monophosphate dehydrogenase (IMPDH)-inhibition activity. Leukotrienes such as leukotriene B_4_ (LTB_4_) are important mediators of asthma, allergy, arthritis, psoriasis, and inflammatory bowel disease [11,12]. Galet*et al.* showed that benzoselenazolinones of type **IV** and the corresponding diselenides **V** dramatically decrease the formation of LTB_4 _[13]. Phenylseleno-substituted pyrimidines of type **VI** exhibit significant inhibitory properties on Urd Pase and TMS. Se-methyl selenocysteine (**VII**) was found to be an antitumor agent, and it has been shown that *β*-elimination reaction is important for this activity [14].

We have a long term interest in selenium chemistry [15,16,17,18,19,20]. Woollins reagent (**WR**, 2,4-bis(phenyl)-1,3-diselenadiphosphetane-2,4-diselenide, [PhP(Se)(µ-Se)]_2_), which is isostructural with the thionation agent [(p-MeOC_6_H_4_)P(S)(µ-S)]_2_ (Lawesson’s reagent), and may be obtained readily from PhPCl_2_, Na_2_Se and Se [21], is an excellent selenation reagent for converting a range of unsaturated organic substrates into unusual phosphorus containing heterocycles [22,23,24,25,26,27,28,29,30,31]. We have recently reported the use of **WR** for organic transformations and for the facile synthesis of primary arylselenoamides from **WR** and ArCN [32]. Although the X-ray crystal structures of some tertiary and secondary selenoamides have been documented [33,34,35,36,37,38,39], surprisingly, no structural information has been published on primary arylselenoamides ArC(Se)NH_2_. We here provide a comparative study of a range of these systems.

## Results and Discussion

Selected metric parameters for compounds **A** - **L** are given in Table 1. The C=Se bond lengths range from 1.822(5) to 1.856(4) Å whilst the C-N bond lengths are in the range 1.270(7) to 1.324(8) Å. This compares with literature values from the Cambridge Database for amide C-Se distances of 1.787-1.885 and C-N of 1.29-1.34 Å. The amide functionality is not particularly coplanar with the aryl backbone, with the selenium atom lying up to 1.406 Å from the aryl ring mean plane with the C(7)-N(7)-Se(7) plane being up to 87^o^ from the aryl mean plane in this case. This maximum deviation from coplanarity may be a function of the presence of two ortho chlorine substituents in compound **L** causing repulsion. However, it is interesting to note that in compound **F** the two independent molecules have quite different degrees of coplanarity for the selenoamide functional group and the aryl ring suggesting that there is little electronic reason for coplanarity.

**Figure 2:**
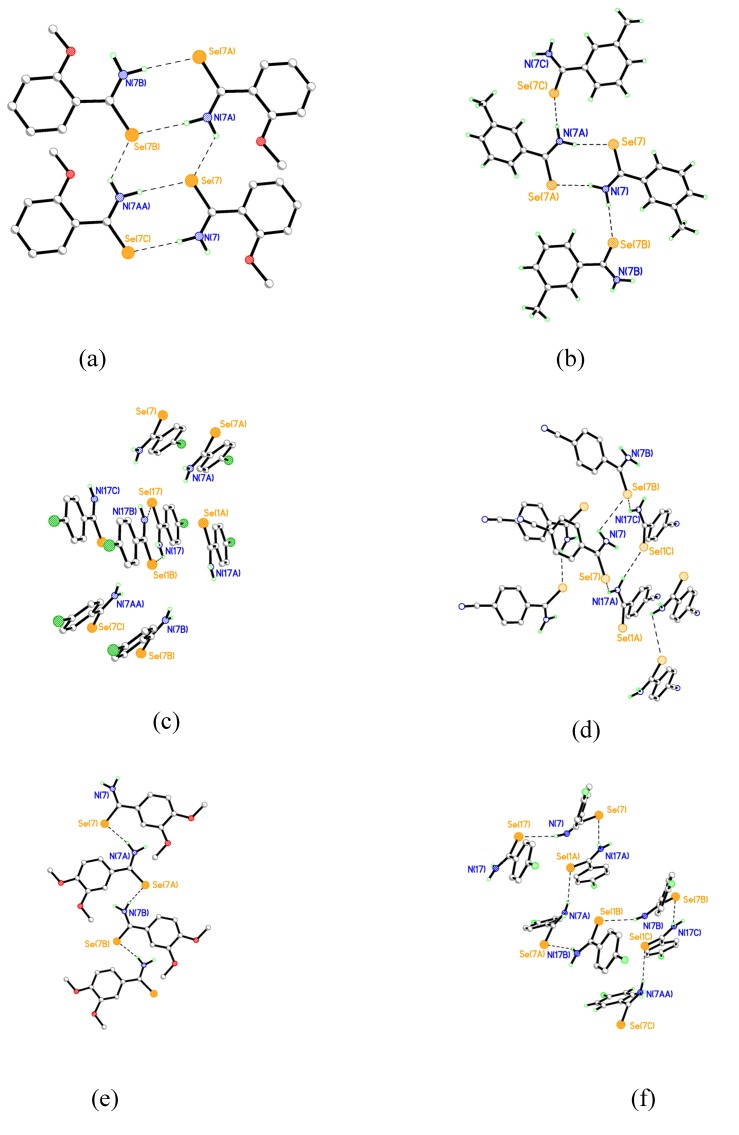
Examples of H-bonding motifs. (a) **1 **ladder (b) **2 **linked dimer (c) **7 **Herring-bone dimers (d) **6 **tetrameric sheets (e) **10 **Chains (f) **9** Helical Chains.

Although hydrogen bonding is well understood for N-H···O and N-H···S systems, there is less data available for N-H···Se systems. We have previously noted that Ph_2_P(Se)NHP(Se)Ph_2_ forms dimers via N-H···Se hydrogen bonds (Se···N 3.19, Se···H 2.52 Å) [40] and it is interesting to note the range of motifs that we have observed in compounds **A** – **L** (Figure 2). We have broadly classified the pattern of hydrogen bonding in compounds **A** - **L** and give selected parameters in Table 2 and provide illustrative examples in Figure 1. It is clear that N-H···Se hydrogen bonding is an important feature of the solid state packing of these molecules and may be a significant influence in biological systems.

**Table 1:** Selected ^77^Se NMR data, bond lengths (Å) and angles (^o^) (Rows containing multiple entries are a.consequence of the presence of more than one independent molecule in the asymmetric unit).

	δ_Se_ (ppm^*^)	C=Se (Å)	C-N (Å)	Aryl/Scene interplanar angle (°)	Deviation of Se from aryl Mean plane (°)
**A**	602.1	1.856(4)	1.311(5)	21	0.469
**B**	641.2	1.848(3)	1.314(3)	36	1.118
**C**	628.6	1.829(7)	1.318(9)	27	0.619
**D**	608.7	1.843(5)	1.316(7)	14	0.343
**E**	579.5	1.848(5)	1.317(6)	17	0.576
**F**	703.7	1.840(11)	1.292(15)	7	0.157
		1.846(14)	1.332(17)	40	1.087
**G**	646.5	1.855(10)	1.310(13)	10	0.173
		1.844(12)	1.295(17)	3	0.104
H*	647.2	1.81(5)	1.27(7)	8-32	0.299 [-0.963]
**I**	629.6	1.829(6)	1.324(8)	37 [18]	1.018 [0.361]
		[1.838(6)]	[1.305(8)]		
**J**	529.0	1.829(6)	1.324(10)	39	0.786
**K**	649.9	1.838(3)	1.317(4)	48	1.346
**L**	715.8	1.822(5)	1.298(7)	87	1.406

*Five independent molecules in the asymmetric unit, average bond lengths and ranges of Se deviations/interplanar angles are given.

**Table 2:** Major N-H···Se hydrogen bonding distances (Å).

	Type	Se···H	Se···N	Se···H-N	Se···H	Se···N	Se···H-N
**A**	Ladder	2.55(1)	3.512(3)	167(3)	2.72(4)	3.403(3)	127(3)
**B**	Linked dimers	2.527(7)	3.489(2)	168(2)	2.539(10)	3.491(2)	164(3)
**C**	Linked dimers	2.59(3)	3.510(6)	156(6)	2.58(3)	3.491(5)	155(5)
**D**	Linked dimers (sheets)	2.55(1)	3.517(4)	170(5)	2.71(5)	3.408(4)	129(4)
**E**	Linked dimers (sheets)	2.55(7)	3.527(4)	174(4)	2.82(5)	3.415(4)	120(4)
**F**	Tetramers (sheets)	2.57(2)	3.58(11)	171(11)	2.63(6)	3.527(11)	152(11)
		2.90(14)	3.430(12)	115(1)	2.69(10)	3.466(10)	136(10)
**G**	Herringbone dimers	2.68(10)	3.502(8)	142(12)	2.85(12)	3.509(8)	125(1)
**H**	Dimers	2.50(17)	3.43(4)	158(4)	2.49(16)	3.43(3)	160(4)
		2.48(9)	3.45(5)	169(5)	2.58(8)	3.43(3)	169(5)
**I**	Helical chain	2.63(3)	3.513(5)	150(4)	2.97(3)	3.628(5)	125(4)
		2.74(4)	3.566(5)	143(5)	2.62(3)	3.512(5)	151(5)
**J**	Chain	2.52(2)	3.483(6)	168(6)			
**K**	Dimers	2.69(1)	3.63(3)	162(3)			
**L**	Linked dimers	2.535(8)	3.511(4)	174(5)	2.579(14)	3.533(4)	165(4)

N-H···O hydrogen bonding: ^a^ H···O 2.09(4) Å, N…O 3.016(7) Å, N-H…O 156(7) Å; ^b^H···O 2.32(3) Å, 2.24(3) Å; N···O 3.076(4), 2.923(3) Å; N-H···O 133(3), 126(3) Å.

## Experimental

**Figure 3:**
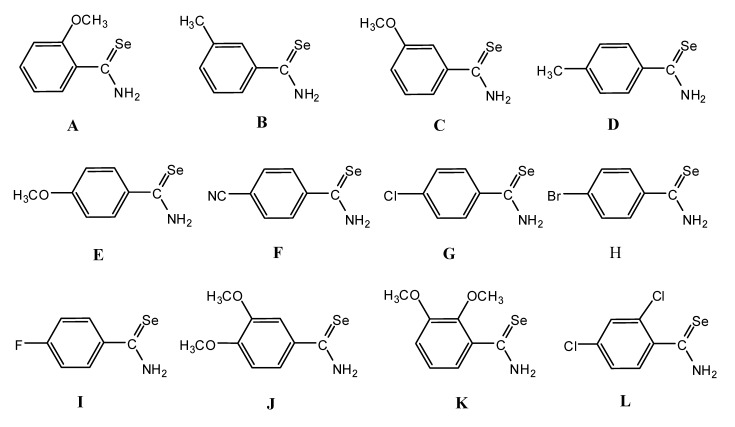
The chemical structures of primary arylselenoamides **A** – **L.**

Primary arylselenoamides **A** – **L** (Figure 3) were prepared as described previously [32]. Their X-ray crystal data (Table 3) were collected at 93 K by using a Rigaku MM007 High brilliance RA generator/confocal optics and Mercury CCD system. Intensities were corrected for Lorentz-polarisation and for absorption. The structures were solved by direct methods. Hydrogen atoms bound to carbon were idealised. Structural refinements were obtained with full-matrix least-squares based on *F^2^* by using the program SHELXTL [41]. CCDC 611494 & 611495 CCDC 713559 - 713568 contain the supplementary crystallographic data for this paper. These data can be obtained free of charge via www.ccdc.cam.ac.uk/conts/retrieving.html or from the Cambridge Crystallographic Data centre, 12 Union Road, Cambridge CB2 1EZ, UK; fax (+44) 1223-336-033; E-mail: deposit@ccdc.cam.ac.uk.

**Table 3:** Details of data collections and refinements for **A** - **L**.

**Compound**	**A**	**B**	**C**	**D**	**E**	**F**
Empirical formula	C_8_H_9_NOSe	C_8_H_9_NSe	C_8_H_9_NOSe	C_8_H_9_NSe	C_8_H_9_NOSe	C_8_H_6_N_2_Se
Crystal color, habit	Yellow, prism	Orange, needle	Orange, prism	Orange, needle	Yellow, platelet	Orange, needle
Crystal dimensions/mm	0.20 × 0.15 × 0.05	0.15 × 0.15 × 0.08	0.20 × 0.20 × 0.10	0.25 × 0.05 × 0.01	0.20 × 0.05 × 0.02	0.30 × 0.06 × 0.03
Crystal system	Monoclinic	Monoclinic	Orthorhombic	Monoclinic	Monoclinic	Orthorhombic
Space group	P2(1) / c	P21(1) / c	Pbca	P21(1) / c	P2(1) / c	P2(1)2(1)2(1)
*a*	5.9600(11)	7.5986(15)	8.4086(17)	9.869(2)	10.108(4)	7.4345(15)
*b*	9.9600(18)	10.464(2)	11.586(2)	6.0039(13)	6.016(2)	6.0647(12)
*c*	14.114(3)	10.163(2)	16.960(4)	13.658(3)	13.765(6)	34.203(6)
*β*	95.506(5)	96.303(6)		105.485(6)	106.907(13)	
*U*/ Å^3^	833.9(3)	803.2(3)	1652.3(6)	779.9(3)	800.9(6)	1542.1(5)
*Z*	4	4	8	4	4	8
*M*	214.1	198.1	214.1	198.1	214.1	209.1
*Dc*/g cm^-3^	1.705	1.638	1.722	1.687	1.776	1.801
*µ*/mm^-1^	4.441	4.595	4.483	4.732	4.625	4.796
*F*(000)	424	392	848	392	424	816
Measured reflections	4447	4308	6679	2899	4170	8341
Independent reflections (R_int_)	1498 (0.0393)	1502 (0.0396)	1377 (0.0908)	1057 (0.0393)	1385 (0.0629)	2715 (0.0562)
Final R1, wR2[I>2σ(I)]	0.0316, 0.0971	0.0269, 0.0680	0.0604, 0.1252	0.0371, 0.0924	0.0458, 0.0911	0.0752, 0.1501
Compound	**G**	**H**	**I**	**J**	**K**	**L**
Empirical formula	C_7_H_6_ClNSe	C_7_H_6_BrNSe	C_7_H_6_FNSe	C_9_H_11_NO_2_Se	C_9_H_11_NO_2_Se	C_9_H_11_NO_2_Se
Crystal color, habit	Orange, prism	Orange, prism	Yellow, prism	Yellow, patelet	Yellow, prism	Yellow, patelet
Crystal dimentions/mm	0.30 × 0.20 × 0.05	0.10 × 0.08 × 0.05	0.30 × 0.20 × 0.10	0.20 × 0.20 × 0.05	0.20 × 0.20 × 0.15	0.20 × 0.20 × 0.05
Crystal system	Triclinic	Triclinic	Monoclinic	Monoclinic	Monoclinic	Monoclinic
Space group	P-1	P-1	C2 / c	Cc	P2(1) /n	Cc
a	4.0219(8)	12.532(3)	31.874(4)	9.905(2)	10.7387(17)	9.905(2)
b	10.774(2)	12.720(3)	3.9871(6)	14.311(3)	7.0242(11)	14.311(3)
c	17.939(4)	17.285(6)	22.322(3)	7.1924(16)	13.539(2)	7.1924(16)
α	92.421(6)	101.69(2)	90	90	90	90
β	92.548(6)	99.83(2)	97.724	104.360(6)	106.225(4)	104.360(6)
γ	91.464(6)	113.572(17)	90	90	90	90
U/ Å^3^	775.6(3)	2373.8(11)	2811.1(7)	987.7(4)	980.6(3)	987.7(4)
Z	4	12	16	4	4	4
M	218.5	263.00	202.1	244.1	244.1	244.1
Dc/g cm^-3^	1.872	2.208	1.910	1.642	1.654	1.642
µ/mm^-1^	5.102	9.713	5.274	3.768	3.796	3.768
F(000)	424	1488	1568	488	488	488
Measured reflections	2011	4531	7115	2686	5199	2686
Independent reflections (R_int_)	1480 (0.0348)	3567 (0.0412)	2503 (0.1074)	1393 (0.0717)	1748 (0.0449)	1393(0.0717)
Final R1, wR2[I>2σ(I)]	0.0728, 0.1853	0.0744, 0.1723	0.0637, 0.1540	0.0431, 0.1250	0.0322, 0.0819	0.0431, 0.1250
